# Regulation of Surgical Procedures and Health Care Facilities Rankings in Nepal

**DOI:** 10.31729/jnma.4088

**Published:** 2019-02-28

**Authors:** Yogesh Acharya, Ranjan Dahal, Navindra Raj Bista, Milan Chandra Khanal, Sangita Bista

**Affiliations:** 1 Avalon University School of Medicine (AUSOM), Willemstad, Curacao, Netherlands Antilles; 2 Saint Peters University Hospital, New Jersey, USA; 3 Tribhuvan University Teaching Hospital, Kathmandu, Nepal; 4 Nepal Medical Association, Kathmandu, Nepal

**Keywords:** *surgical procedures*, *health care facilities*, *safety*, *surgery*, *WHO*

## Abstract

Globally, millions of surgeries are performed each year to compliment and manage a diverse set of medical conditions. Adverse surgical outcomes constitute a major proportion of avoidable death and disabilities in the hospital, especially in low-income countries like Nepal. A comprehensive study on the standards of surgical procedures and its institutional regulations is missing. We discuss here the importance of surgical regulation based on it's financial as well as healthcare implications in the Nepalese healthcare system.

## INTRODUCTION

Surgical safety is considered to be a global health indicator due to the high perioperative complications.^1^ Adverse surgical outcomes constitute a major proportion of an avoidable deaths and disabilities in the hospital, especially in low income countries like Nepal.^2^ Despite global health importance, they are not effectively regulated and there is a definitive lack of authoritative bodies to standardize and validate them in many parts of the world. These situations resonate in Nepal as we have no authorized governmental or non-governmental health bodies to regulate and standardize the surgical procedures nationally, except some local committees at the institutional level. Many of the ongoing surgical skills have been passed down through generations by professors in medical colleges and universities with considerable individual variations. The systematic studies of these variations have not been performed and their perceived beneficence is yet to be proven. Although these practices resonate with other countries (Food and Drug Administration regulate the drug for safety and efficacy in the US but not the ongoing surgical practices and innovations), the adverse financial and healthcare implications due to unregulated surgeries among patients in low resource countries like Nepal can be overwhelming.^3^ Although hospital ranking system is absent in Nepal, adoption of a similar grading system can possibly increase adherence to safety standards and lower adverse medical and surgical outcome.

## BACKGROUND

Surgical procedures are an integral component of patient care. An estimated 234 million surgeries^1^ are performed each year globally in various situations and settings, ranging from simple incision and drainage to complex and technically challenging procedures like organ transplant. World Health Organization (WHO) and World Bank have acknowledged the importance of surgery as a predictor of global health upliftment and highlights its relevance in the prevention of a significant proportion of disability in the resource limited settings.^4^ We discuss here the importance of surgical regulation in the context of Nepalese health care system.

WHO^5^ introduced 19 items surgical safety checklist and performed a study in diverse economic circumstances and populations to witness its impact in reduction of operative mortality and showed a significant decrement in death after the introduction of the checklist. This checklist clearly demonstrated an improvement in patient safety and decline in adverse surgical outcomes.^6^ We recommend either the use of this relatively simple and inexpensive WHO surgical safety checklist or formulation of a customized local checklist system that is best suited for the given institutional settings.

Hospital ranking system can be a suitable alternative to grade the hospital on the basis of its safety performance. Hospitals in the US^7^ are ranked in 16 specialty areas, out of which 12 specialty ranking is determined mostly by the composite data system related to the medical and surgical outcome. These parameters used for hospitals performance are based on Centers for Medicare and Medicaid Services (CMS) and includes: surgical site infection, death and unplanned readmission within a month, revision surgery within a year, length of stay and discharge, expert opinion from board certified physicians, and other service related indicators.

## WAY FORWARD

Surgical variations are common in standard practice and necessitate the specific need for patient centered intervention. The efficacy of these variations is yet not proven, and many of them go unnoticed. Unfortunately, there are no regulatory bodies to oversee the existing and rapidly increasing healthcare institutions with established surgical setups in Nepal. Distinct lack of essential resources and equipment, technical expertise, economic limitations, poor organizational management, practical difficulty in conducting standard study, and ideological preference of a particular surgical procedure can be plausible answers.^8^ These explanations highlight an importance of a comprehensive study to point out possible limitations, as we might still be missing many important links due to paucity of the available evidences.

Nevertheless, widening gaps in effectiveness and safety of these procedures can invariably have a major impact in our healthcare expenditure and progressively worsen struggling health care setups in the country. It is pertinent that medical procedure be well regulated and practiced in a safe environment. For this, we recommend all the key stakeholders, including government, non government bodies, practicing physicians, and other health care personnel to come together, discuss, and carefully formulate a specific plan focused on standardization and effective regulation of surgical procedures across the country. Similarly, we advocate the use of WHO surgical safety checklist, until there is a provision of a standard checklist suitable for the local context. Ranking health care facilities based on their surgical performance will provide more flexibility to the patients and can serve as a suitable alternative to promote safety standards.

It is necessary to improve surgical process to uplift the quality of patient care and ensure surgical safety. Beyond doubt, proper management of surgical complications can prevent thousands of deaths globally. There are two major surgical regulations system that can be adopted systematically across all the surgical institutions in Nepal based on their relevance and feasibility.

## Conflict of Interest

**None.**
